# 9,10-Diiodo­phenanthrene

**DOI:** 10.1107/S1600536812045758

**Published:** 2012-11-10

**Authors:** Ruri Yokota, Chitoshi Kitamura, Takeshi Kawase

**Affiliations:** a Department of Materials Science and Chemistry, Graduate School of Engineering, University of Hyogo, 2167 Shosha, Himeji, Hyogo 671-2280, Japan

## Abstract

The whole mol­ecule of the title compound, C_14_H_8_I_2_, is generated by crystallographic twofold symmetry. The mol­ecule is planar [maximum deviation = 0.0323 (6) Å] with the I atoms displaced from the mean plane of the phenanthrene ring system by only 0.0254 (5) Å. In the crystal, mol­ecules form face-to-face slipped anti­parallel π–π stacking inter­actions along the *c* axis with an inter­planar distance of 3.499 (7) Å.

## Related literature

For the synthesis of the title compound, see: Rodrígeuz-Lojo *et al.* (2012 ▶). For a related structure, see: Yokota *et al.* (2012 ▶).
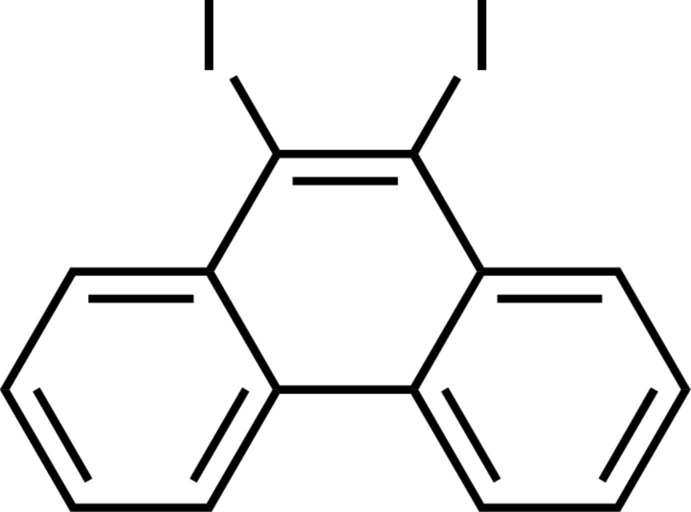


## Experimental

### 

#### Crystal data

C_14_H_8_I_2_
*M* *_r_* = 430
Monoclinic, 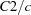

*a* = 18.094 (2) Å
*b* = 9.4557 (14) Å
*c* = 7.4187 (10) Å
β = 111.953 (3)°
*V* = 1177.2 (3) Å^3^
*Z* = 4
Mo *K*α radiation
μ = 5.31 mm^−1^
*T* = 223 K
0.52 × 0.08 × 0.05 mm

#### Data collection

Rigaku R-AXIS RAPID diffractometer
Absorption correction: numerical (*NUMABS*; Higashi, 1999 ▶) *T*_min_ = 0.388, *T*_max_ = 0.869
5595 measured reflections
1345 independent reflections
1077 reflections with *I* > 2σ(*I*)
*R*_int_ = 0.030

#### Refinement

*R*[*F* ^2^ > 2σ(*F* ^2^)] = 0.033
*wR*(*F* ^2^) = 0.083
*S* = 1.12
1345 reflections
73 parameters
H-atom parameters constrained
Δρ_max_ = 0.92 e Å^−3^
Δρ_min_ = −0.92 e Å^−3^

### 

Data collection: *RAPID-AUTO* (Rigaku, 1999 ▶); cell refinement: *PROCESS-AUTO* (Rigaku, 1998 ▶); data reduction: *PROCESS-AUTO*; program(s) used to solve structure: *SIR2004* (Burla *et al.*, 2005 ▶); program(s) used to refine structure: *SHELXL97* (Sheldrick, 2008 ▶); molecular graphics: *ORTEP-3 for Windows* (Farrugia, 1997 ▶); software used to prepare material for publication: *WinGX* (Farrugia, 1999 ▶).

## Supplementary Material

Crystal structure: contains datablock(s) global, I. DOI: 10.1107/S1600536812045758/jj2155sup1.cif

Structure factors: contains datablock(s) I. DOI: 10.1107/S1600536812045758/jj2155Isup2.hkl

Supplementary material file. DOI: 10.1107/S1600536812045758/jj2155Isup3.cml

Additional supplementary materials:  crystallographic information; 3D view; checkCIF report

## Figures and Tables

**Fig. 1.**
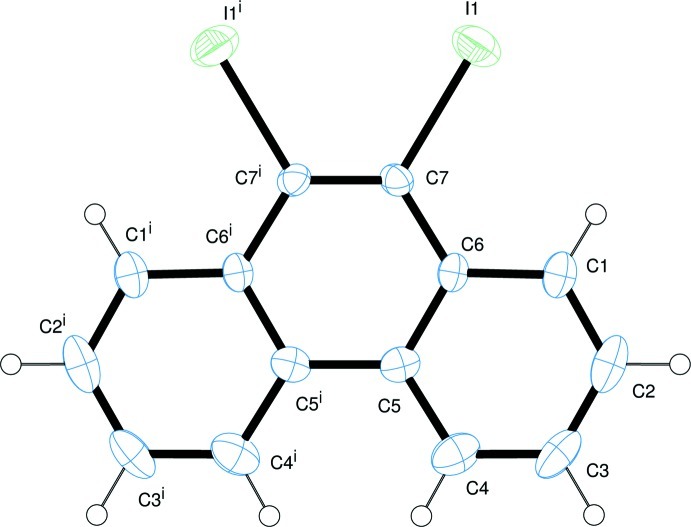
The molecular structure of the title compound, showing the atomic numbering and 40% probability displacement ellipsoids. Symmetry code: (i) -x + 1, y, -z + 1.5.

**Fig. 2.**
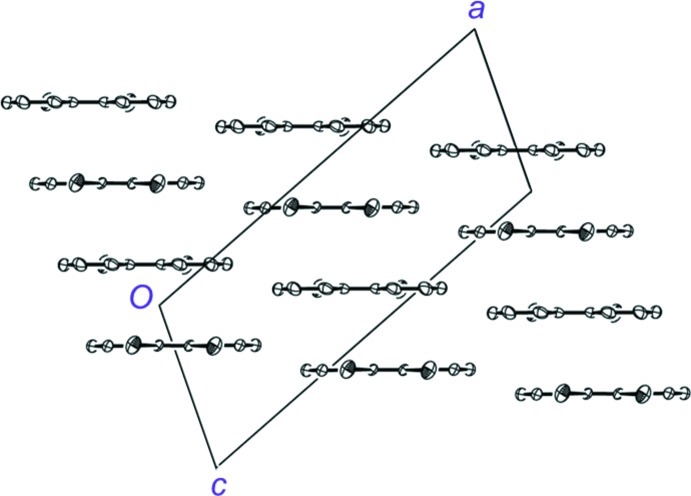
The packing diagram of the title compound viewed along the b axis. Hydrogen atoms have been omitted for clarity.

## supplementary crystallographic information

### Comment

*o*-Diiodoarenes are valuable synthetic intermediates. We were able to obtain suitable single crystals of 9,10-diiodoophenanthrene, the title compound, by the recently published synthetic method of Rodrígeuz-Lojo *et al.* (2012). We report herein the crystal structure of C_14_H_8_I_2_, the title compound.

In the molecular structure of the title compound, (I), the mean plane of the arene ring displays a maximum deviation of 0.0323 (6) Å for I1 (Fig. 1). The molecule possesses *C*_2_ symmetry, and half of the formula unit is crystallographically independent. Bonds lengths and angles are in good agreement with the standard values. Crystal packing is stabilized by face-to-face, slipped, antiparrallel, π-π stacking along the direction of the *c* axis with an interplanar distance of 3.499 (7) Å (Fig. 2). Very recently, we have reported the crystal structure of 9,10-dibromophenanthrene (Yokota *et al.*, 2012), the bromine analog of (I), which displays a similar packing arrangement.

### Experimental

The title compound was prepared according to the literature method (Rodrígeuz-Lojo *et al.*, 2012) Single crystals suitable for X-ray analysis were obtained from a toluene-hexane solution.

### Refinement

All the aromatic H atoms were positioned geometrically and refined using a riding model with C—H = 0.94 Å and *U*_iso_(H) = 1.2*U*_eq_(C).

### Crystal data

**Table:**

C_14_H_8_I_2_	*F*(000) = 792
*M**_r_* = 430	*D*_x_ = 2.426 Mg m^−^^3^
Monoclinic, *C*2/*c*	Mo *K*α radiation, λ = 0.71073 Å
Hall symbol: -C 2yc	Cell parameters from 3465 reflections
*a* = 18.094 (2) Å	θ = 3.1–27.5°
*b* = 9.4557 (14) Å	µ = 5.31 mm^−^^1^
*c* = 7.4187 (10) Å	*T* = 223 K
β = 111.953 (3)°	Needle, colorless
*V* = 1177.2 (3) Å^3^	0.52 × 0.08 × 0.05 mm
*Z* = 4	

### Data collection

**Table:**

Rigaku R-AXIS RAPID diffractometer	1345 independent reflections
Radiation source: fine-focus sealed x-ray tube	1077 reflections with *I* > 2σ(*I*)
Graphite monochromator	*R*_int_ = 0.030
Detector resolution: 10 pixels mm^-1^	θ_max_ = 27.5°, θ_min_ = 3.5°
ω scans	*h* = −23→22
Absorption correction: numerical (*NUMABS*; Higashi, 1999)	*k* = −12→12
*T*_min_ = 0.388, *T*_max_ = 0.869	*l* = −8→9
5595 measured reflections	

### Refinement

**Table:**

Refinement on *F*^2^	Primary atom site location: structure-invariant direct methods
Least-squares matrix: full	Hydrogen site location: inferred from neighbouring sites
*R*[*F*^2^ > 2σ(*F*^2^)] = 0.033	H-atom parameters constrained
*wR*(*F*^2^) = 0.083	*w* = 1/[σ^2^(*F*_o_^2^) + (0.0077*P*)^2^ + 13.1956*P*] where *P* = (*F*_o_^2^ + 2*F*_c_^2^)/3
*S* = 1.12	(Δ/σ)_max_ < 0.001
1345 reflections	Δρ_max_ = 0.92 e Å^−^^3^
73 parameters	Δρ_min_ = −0.92 e Å^−^^3^
0 restraints	

### Special details

**Table:**

Geometry. All s.u.'s (except the s.u. in the dihedral angle between two l.s. planes) are estimated using the full covariance matrix. The cell s.u.'s are taken into account individually in the estimation of s.u.'s in distances, angles and torsion angles; correlations between s.u.'s in cell parameters are only used when they are defined by crystal symmetry. An approximate (isotropic) treatment of cell s.u.'s is used for estimating s.u.'s involving l.s. planes.
Refinement. Refinement of *F*^2^ against ALL reflections. The weighted *R*-factor *wR* and goodness of fit *S* are based on *F*^2^, conventional *R*-factors *R* are based on *F*, with *F* set to zero for negative *F*^2^. The threshold expression of *F*^2^ > 2σ(*F*^2^) is used only for calculating *R*-factors(gt) *etc*. and is not relevant to the choice of reflections for refinement. *R*-factors based on *F*^2^ are statistically about twice as large as those based on *F*, and *R*- factors based on ALL data will be even larger.

### Fractional atomic coordinates and isotropic or equivalent isotropic displacement parameters (Å^2^)

**Table:**

	*x*	*y*	*z*	*U*_iso_*/*U*_eq_	
C1	0.3399 (3)	0.4417 (6)	0.4793 (7)	0.0368 (12)	
H1	0.3127	0.3564	0.4332	0.044*	
C2	0.3020 (3)	0.5667 (8)	0.4155 (8)	0.0470 (15)	
H2	0.2488	0.5669	0.3275	0.056*	
C3	0.3408 (3)	0.6927 (7)	0.4781 (9)	0.0467 (15)	
H3	0.3146	0.7786	0.4306	0.056*	
C4	0.4171 (4)	0.6931 (6)	0.6089 (8)	0.0424 (13)	
H4	0.4426	0.78	0.6529	0.051*	
C5	0.4587 (3)	0.5675 (5)	0.6798 (7)	0.0289 (10)	
C6	0.4192 (3)	0.4383 (5)	0.6137 (7)	0.0261 (9)	
C7	0.4618 (3)	0.3079 (5)	0.6846 (7)	0.0281 (10)	
I1	0.40244 (3)	0.11933 (4)	0.58098 (7)	0.05783 (18)	

### Atomic displacement parameters (Å^2^)

**Table:**

	*U*^11^	*U*^22^	*U*^33^	*U*^12^	*U*^13^	*U*^23^
C1	0.029 (2)	0.051 (3)	0.029 (3)	0.003 (2)	0.008 (2)	0.001 (2)
C2	0.026 (3)	0.078 (4)	0.034 (3)	0.012 (3)	0.007 (2)	0.010 (3)
C3	0.038 (3)	0.057 (4)	0.048 (3)	0.024 (3)	0.019 (3)	0.020 (3)
C4	0.048 (3)	0.040 (3)	0.048 (3)	0.012 (2)	0.028 (3)	0.008 (3)
C5	0.030 (2)	0.030 (2)	0.031 (3)	0.0021 (19)	0.017 (2)	0.0012 (19)
C6	0.023 (2)	0.035 (2)	0.024 (2)	0.0010 (18)	0.0126 (18)	0.0001 (18)
C7	0.033 (2)	0.026 (2)	0.025 (2)	−0.0029 (18)	0.010 (2)	−0.0020 (18)
I1	0.0670 (3)	0.0402 (2)	0.0526 (3)	−0.01527 (19)	0.0067 (2)	−0.00726 (18)

### Geometric parameters (Å, º)

**Table:**

C1—C2	1.359 (8)	C4—C5	1.399 (7)
C1—C6	1.409 (7)	C4—H4	0.94
C1—H1	0.94	C5—C6	1.408 (7)
C2—C3	1.372 (9)	C5—C5^i^	1.467 (10)
C2—H2	0.94	C6—C7	1.445 (6)
C3—C4	1.359 (8)	C7—C7^i^	1.360 (9)
C3—H3	0.94	C7—I1	2.075 (5)
			
C2—C1—C6	120.9 (5)	C5—C4—H4	119.1
C2—C1—H1	119.6	C4—C5—C6	118.2 (5)
C6—C1—H1	119.6	C4—C5—C5^i^	121.9 (3)
C1—C2—C3	120.7 (5)	C6—C5—C5^i^	119.8 (3)
C1—C2—H2	119.7	C5—C6—C1	118.5 (5)
C3—C2—H2	119.7	C5—C6—C7	118.7 (4)
C4—C3—C2	119.9 (5)	C1—C6—C7	122.8 (5)
C4—C3—H3	120.1	C7^i^—C7—C6	121.5 (3)
C2—C3—H3	120.1	C7^i^—C7—I1	120.77 (13)
C3—C4—C5	121.8 (6)	C6—C7—I1	117.8 (3)
C3—C4—H4	119.1		
			
C6—C1—C2—C3	1.1 (8)	C5^i^—C5—C6—C7	−0.9 (8)
C1—C2—C3—C4	−1.6 (9)	C2—C1—C6—C5	−0.4 (7)
C2—C3—C4—C5	1.4 (9)	C2—C1—C6—C7	−179.6 (5)
C3—C4—C5—C6	−0.7 (8)	C5—C6—C7—C7^i^	1.2 (8)
C3—C4—C5—C5^i^	179.7 (6)	C1—C6—C7—C7^i^	−179.6 (6)
C4—C5—C6—C1	0.2 (7)	C5—C6—C7—I1	−179.0 (3)
C5^i^—C5—C6—C1	179.8 (5)	C1—C6—C7—I1	0.2 (6)
C4—C5—C6—C7	179.5 (5)		

Symmetry code: (i) −*x*+1, *y*, −*z*+3/2.
